# The Contribution of EDF1 to PPARγ Transcriptional Activation in VEGF-Treated Human Endothelial Cells

**DOI:** 10.3390/ijms19071830

**Published:** 2018-06-21

**Authors:** Alessandra Cazzaniga, Laura Locatelli, Sara Castiglioni, Jeanette Maier

**Affiliations:** Dipartimento di Scienze Biomediche e Cliniche L. Sacco, Università degli Studi di Milano, I-20157 Milan, Italy; alessandra.cazzaniga@unimi.it (A.C.); laura.locatelli@unimi.it (L.L.); sara.castiglioni@unimi.it (S.C.)

**Keywords:** endothelial cells, vascular endothelial growth factor, Peroxisome proliferator-activated receptor γ, Endothelial Differentiation-related factor 1

## Abstract

Vascular endothelial growth factor (VEGF) is important for maintaining healthy endothelium, which is crucial for vascular integrity. In this paper, we show that VEGF stimulates the nuclear translocation of endothelial differentiation-related factor 1 (EDF1), a highly conserved intracellular protein implicated in molecular events that are pivotal to endothelial function. In the nucleus, EDF1 serves as a transcriptional coactivator of peroxisome proliferator-activated receptor gamma (PPARγ), which has a protective role in the vasculature. Indeed, silencing *EDF1* prevents VEGF induction of PPARγ activity as detected by gene reporter assay. Accordingly, silencing *EDF1* markedly inhibits the stimulatory effect of VEGF on the expression of *FABP4*, a PPARγ-inducible gene. As nitric oxide is a marker of endothelial function, it is noteworthy that we report a link between *EDF1* silencing, decreased levels of *FABP4*, and nitric oxide production. We conclude that EDF1 is required for VEGF-induced activation of the transcriptional activity of PPARγ.

## 1. Introduction

Peroxisome proliferator-activated receptor gamma (PPARγ) is a ubiquitous ligand-inducible transcription factor belonging to the nuclear receptor superfamily [1]. PPARγ, which is highly expressed in adipose tissue, is the master regulator of adipocyte differentiation and is fundamental for mature adipocyte function [2,3,4]. PPARγ is also implicated in glucose homeostasis as it upregulates genes involved in glucose uptake and controls the expression of adipokines, thereby having an effect on insulin sensitivity [3,5,6]. It is now clear that PPARγ plays an important protective role in the vasculature. Its activity has been proven both in smooth muscle cells, where it has a role in the regulation of vascular tone [7], and in endothelial cells (EC), where it exerts anti-inflammatory and antioxidant effects [8]. In endothelial-specific PPARγ^−/−^ mice, loss of PPAR𝛾 contributes to endothelial dysfunction associated with enhanced production of free radicals and exacerbated inflammation [9]. Accordingly, human and animal studies indicate that thiazolidinediones (TZD), which are largely utilized as antidiabetic drugs and PPARγ activators, attenuate vascular diseases including atherosclerosis [10,11]. Post-translational modifications—including phosphorylation, acetylation, and sumoylation—are important in carving PPARγ-driven gene expression [12]. Another layer of control over PPARγ activity depends on its interactions with coactivators and corepressors [12], which regulate transcriptional activity by reshaping chromatin structure via histone deacetylases and histone acetyltransferases [13]. Indeed, upon activation by small natural lipophilic ligands or synthetic agonists, the conformation of PPARγ changes—corepressors are released and coactivators are recruited [3]—thus resulting in transcriptional activation.

Endothelial differentiation-related factor 1 (EDF1), a highly conserved intracellular protein of 148 amino acids, has been identified as one of PPARγ’s coactivators [14,15]. Initially, the role of EDF1 as a transcriptional coactivator was described in the silkworm *Bombyx mori* and in *Drosophila melanogaster* where EDF1 stimulates the activity of the FTZ-F1 nuclear receptor [16]. At the time, EDF1 was demonstrated to serve as a coactivator for several transcription factors [17,18]. This included some nuclear receptors implicated in lipid metabolism, such as steroidogenic factor 1, liver receptor homologue 1, liver X receptor α and, as mentioned above, PPARγ [14,15]. In particular, in 3T3-L1 preadipocytes, EDF1 is required for PPARγ-mediated differentiation and gene expression programs [15]. In human macrovascular EC EDF1 was described as a factor implicated in differentiation and spatial organization [19]. In these cells EDF1 is localized mainly in the cytosol where it binds calmodulin [20] under basal conditions. In response to various stimuli, it is translocated to the nucleus where it interacts with the TATA box-binding protein [20].

Apart from its pivotal role in vasculogenesis and angiogenesis, VEGF is essential for endothelial polarity and survival, thus contributing to the integrity of mature vessels [21]. This issue is relevant since ECs are key players in organogenesis as well as in promoting adult organ maintenance. To this purpose, it is noteworthy that VEGF is a critical component of the cross-talk between organs and tissues and the vessels [21].

For this study, we considered three known facts: (1) PPARγ ligands influence VEGF action [8]; (2) PPARγ contributes to maintain normal vascular function [7,8,9,10,11]; and (3) EDF1 modulates the activity of PPARγ [14,15]. We used these factors to investigate whether EDF1 acts as a regulator of PPARγ activity in human macrovascular EC under normal culture conditions and after treatment with VEGF.

## 2. Results

### 2.1. Translocation of EDF1 to the Nucleus in Response to VEGF

**Figure 1:**
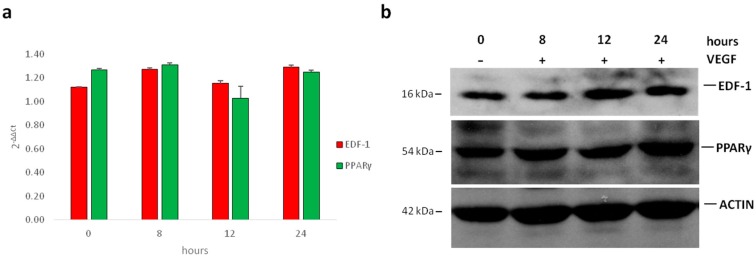
The total amounts of endothelial differentiation-related factor 1 (EDF1) and peroxisome proliferator-activated receptor gamma (PPARγ) in cells treated with VEGF. Human umbilical vein endothelial cells (HUVEC) were treated with 50 ng/mL of vascular endothelial growth factor (VEGF) for 0, 8, 12, and 24 h. (**a**) Real-Time PCR was performed on RNA samples. Two different experiments in triplicate were performed; (**b**) cell lysates were analyzed by western blot using antibodies against EDF1, PPARγ, and actin. A representative blot is shown.

**Figure 2:**
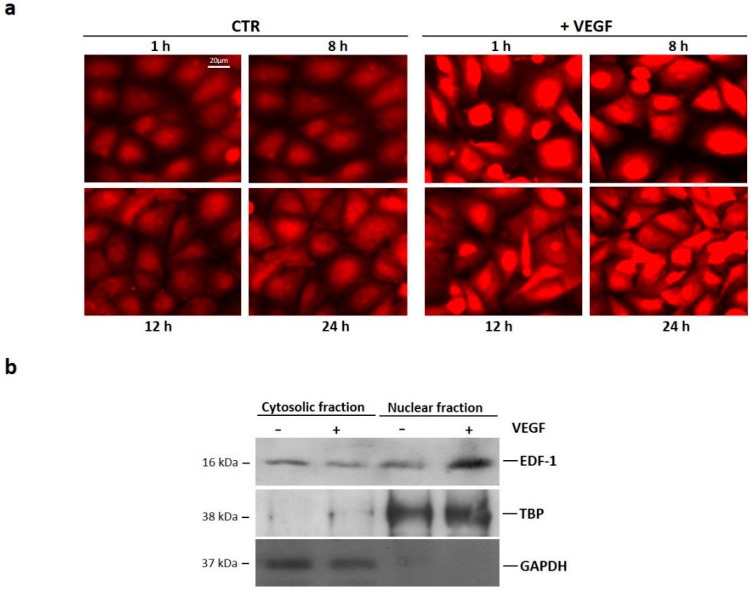
Subcellular localization of EDF1 in cells treated with VEGF. (**a**) HUVEC were treated with VEGF (50 ng/mL) for 1, 8, 12, and 24 h. Immunofluorescence was performed using anti-EDF1 immunopurified immunoglobulin G (IgGs) and rhodamine-conjugated anti-rabbit IgGs; (**b**) HUVEC were treated with VEGF (50 ng/mL) for 1 h. Western blot was performed on nuclear and cytosolic fractions using antibodies against EDF1. GAPDH and TBP were used as cytosolic and nuclear markers, respectively. A representative blot is shown.

Initially, we evaluated whether VEGF modulates the total amounts of EDF1 and PPARγ in human umbilical vein endothelial cells (HUVEC). Confluent cells were treated with VEGF (50 ng/mL) for different times. We performed Real-Time PCR as well as western blot analysis and found no modulation in the levels of EDF1 and PPARγ after 8, 12, and 24 h exposure to VEGF (Figure 1 and Supplementary S1). Because EDF1 translocates to the nucleus when HUVEC are stimulated with the phorbol ester 12-O-Tetradecanoylphorbol-13-acetate (TPA) or with forskolin [20,22], we evaluated the subcellular localization of EDF1 in cells treated with VEGF (50 ng/mL) for different times. By immunofluorescence, EDF1 was detectable both in the cytosol and in the nucleus of unstimulated cells. After being treated with VEGF, EDF1 accumulated in the nuclei after 1 h and remained nuclear-associated for the following 24 h (Figure 2a and Supplementary S2a). Western blot on nuclear and cytosolic fractions isolated after 1 h treatment with VEGF confirmed these results (Figure 2b and Supplementary S2b).

### 2.2. Interaction between EDF1 and PPARγ in HUVEC

**Figure 3:**
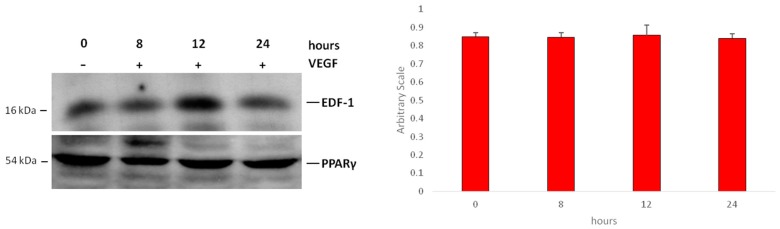
The interaction between EDF1 and PPARγ in HUVEC treated with VEGF. HUVEC were treated with VEGF (50 ng/mL) for different times. Cell lysates were immunoprecipitated with monoclonal antibodies against PPARγ and analyzed by western blot using rabbit antibodies against EDF1 (**upper panel**). The filter was then probed with rabbit anti-PPARγ antibodies to verify the equal amounts of immunoprecipitated proteins (**lower panel**). Densitometric analysis was performed using ImageJ software. EDF1/PPARγ ratio was calculated on three blots from separate experiments ± standard deviation.

We evaluated the interaction between EDF1 and PPARγ in HUVEC treated with VEGF (50 ng/mL) for various times. Cell lysates were immunoprecipitated with antibodies against PPARγ. Western blot was performed on the immunoprecipitates to detect EDF1. EDF1 and PPARγ interacted in nonstimulated cells and VEGF did not significantly modulate this interaction at the time points tested (Figure 3 and Supplementary S3).

### 2.3. Effect of Silencing EDF1 in VEGF-Induced PPARγ Activity

**Figure 4:**
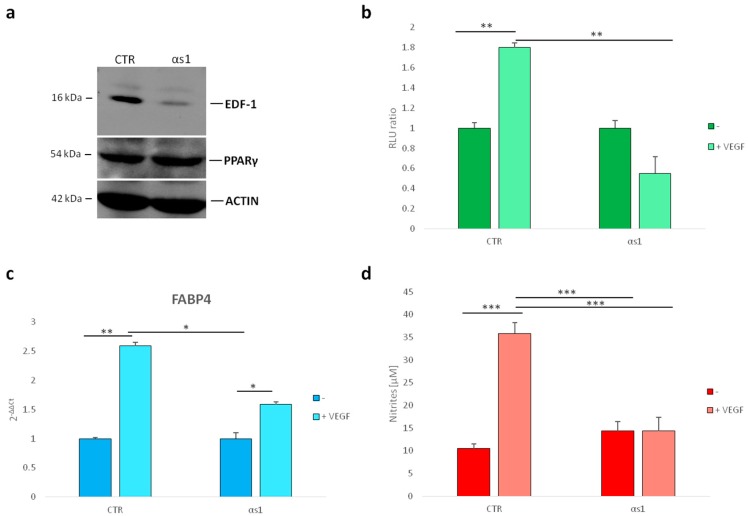
PPARγ transcriptional activity in HUVEC with silenced EDF1. (**a**) The modulation of PPARγ was evaluated in αs1 cells (αs1) (HUVEC with stably silenced EDF1) and compared to HUVEC transfected with a scrambled nonsilencing sequence (used as control) (CTR). Cell lysates were analyzed by western blot using antibodies against EDF1, PPARγ, and actin. A representative blot is shown; (**b**) PPARγ activity was evaluated by luciferase assay in αs1 cells and compared to the control HUVEC; (**c**) Real-Time PCR was performed on RNA samples from αs1 cells and relative control, treated or not with VEGF (50/ng/mL) for 24 h. Three different experiments in triplicate were performed; (**d**) Nitric oxide (NO) release was measured using the Griess method for nitrate quantification. The values were expressed as the mean of three different experiments in triplicate ± standard deviation. * *p* < 0.05, ** *p* < 0.01, *** *p* < 0.001.

To study if EDF1 contributes to PPARγ transcriptional activity in VEGF-treated HUVEC, we utilized HUVEC with stably silenced EDF1, denominated αs1 cells [23]. We used HUVEC transfected with a nonsilencing sequence [23] as the control (CTR). It is noteworthy that PPARγ did not change in αs1 cells compared to their controls as demonstrated by western blot (Figure 4a and Supplementary S4).

We then transfected subconfluent αs1 cells and the control cells with a vector expressing luciferase under the control of a PPARγ responsive consensus (pDR1) [24]. After 4 h, the cells were treated with VEGF (50 ng/mL) and luciferase activity was measured after 24 h. While VEGF stimulated PPARγ transcriptional activation in control cells, this effect was prevented by silencing *EDF1* (Figure 4b). To reinforce this finding, we analyzed the expression of a PPARγ downstream target gene, i.e., fatty acid-binding protein 4 (FABP4), which is known to be upregulated in HUVEC after 24 h exposure to VEGF [25]. We cultured HUVEC in the presence of VEGF (50 ng/mL) for 24 h. Using Real-Time PCR, we confirmed the overexpression of *FABP4* RNA in control HUVEC treated with VEGF. In αs1 cells, which downregulate EDF1, the induction was significantly reduced (Figure 4c).

Because HUVEC with silenced FABP4 produce lower amounts of nitric oxide (NO) than controls and are insensitive to the stimulatory effect of VEGF [26], we measured the release of NO in αs1 cells and their controls that were treated or not with VEGF for 24 h. Figure 4d shows that while VEGF induced NO secretion in control cells, it did not exert any significant effect in αs1 cells.

## 3. Discussion

ECs line the inner face of blood vessels and their integrity is fundamental for vascular homeostasis and circulatory function [27]. Indeed, ECs are implicated in maintaining blood fluidity, governing leukocyte trafficking and vascular tone, and in regulating immune response. Consequently, it is not surprising that endothelial dysfunction, which is characterized by a pro-oxidant and pro-inflammatory phenotype, orchestrates events leading to cardiovascular diseases. Moreover, healthy endothelial cells are crucial for the maintenance of normal energy metabolism and, therefore, physiologic function of all tissues [27]. There is now evidence that PPARγ is a key regulator of endothelial function [21,27] and, accordingly, PPARγ activators inhibit the expression of proinflammatory molecules and the synthesis of free radicals [27]. Many factors contribute to the integrity of the endothelium including VEGF, which is critical for endothelial survival and barrier function in mature vessels [21]. On these bases, we investigated whether VEGF activates PPARγ in HUVEC by recruiting the transcriptional co-activator EDF1. We found that while VEGF does not change the total amounts of EDF1, it rapidly induces EDF1 nuclear translocation, which is maintained for 24 h. These results are in accordance with previous data showing EDF1 nuclear accumulation in HUVEC treated with the phorbol ester 12-O-Tetradecanoylphorbol-13-acetate (TPA) and the forskolin, which raise intracellular cAMP [20,23]. Interestingly, TPA, forskolin, and VEGF all induce the phosphorylation of EDF1 [20,22,23], and we hypothesize that phosphorylation has a role in increasing the nuclear accumulation of EDF1. It should be noted that EDF1 does not have a nuclear targeting sequence, and it is likely that a shuttle protein drives EDF1 to the nucleus. If this is the case, we postulate that VEGF enhances this shuttle mechanism. In the nucleus, VEGF stimulates the transcriptional activity of PPARγ in an EDF1-dependent manner. Indeed, silencing EDF1 prevents VEGF-induced PPARγ activity. Since VEGF increases the transcriptional activity of PPARγ in endothelial cells without altering its interaction with EDF1, it is possible that VEGF induces a specific PPARγ ligand or inhibits a corepressor, thus altering the balance between various transcriptional corepressors and coactivators. These results highlight an important difference between adipocytes and endothelial cells. In 3T3-L1 preadipocytes, silencing *EDF1* decreases the total amounts of PPARγ and, in parallel, its transcriptional activity [15]. By contrast, in HUVEC, silencing *EDF1* affects only its transcriptional activity.

We also evaluated the expression of a PPARγ-responsive gene in HUVEC with silenced EDF1. In particular, we focused on the modulation of *FABP4*, which encodes a cytoplasmic protein that has a role in endothelial fatty acid metabolism and free radical production. It also impairs proliferation and sprout elongation and impacts on nitric oxide synthesis [25,28]. Interestingly, pioglitazone—an insulin-sensitizing thiazolidinedione and a PPARγ agonist—increases FABP4 levels. In this study, we show that the activation of PPARγ by VEGF induces *FABP4* expression through the involvement of EDF1. Indeed, silencing *EDF1* markedly inhibits the stimulatory effect of VEGF on *FABP4* expression. It is known that VEGF induces *FABP4* through the Delta-like ligand (DLL) 4/NOTCH1 pathway [25]. Our results indicate that also PPARγ contributes to VEGF induction of *FABP4* in HUVEC.

NO is a pivotal mediator of VEGF-induced responses and is essential for vascular function [29]. The regulation of NO production is very complex and it is noteworthy that both PPARγ and FABP4 are involved. Indeed, in endothelial cells, the activation of PPARγ increases NO release [30] while the downregulation of *FABP4* reduces it [28]. To this purpose, we show that αs1 cells do not respond to VEGF by increasing NO release. We hypothesize that the downregulation of EDF1 impairs VEGF-induced activation of PPARγ with consequent reduction of *FABP4* and NO synthesis.

We conclude that VEGF induces EDF1 translocation to the nucleus where it acts as a transcriptional coactivator of PPARγ. The transcriptional activation of PPARγ increases the expression of FABP4, which is known to regulate NO production. Because NO is a marker of endothelial function, our results substantiate that PPARγ activation has a role in maintaining the integrity of vessels and highlight EDF1 as a novel player in the complex regulation of PPARγ transcriptional activity in the endothelium. On these bases, we propose that EDF1 makes an important contribution to maintain endothelial integrity, and this may be crucial in the prevention of cardiovascular diseases.

## 4. Materials and Methods

### 4.1. Cell Culture

HUVEC—widely accepted as a model of macrovascular EC—were obtained from the American Type Culture Collection (ATCC) and cultured in M199 containing 10% fetal bovine serum, 1 mM glutamine, endothelial cell growth factor (150 µg/mL), 1 mM sodium pyruvate and heparin (5 units/mL) on 2% gelatin-coated dishes [20]. In some experiments, we utilized HUVEC stably transfected to silence *EDF1* (αs1), while their controls (CTR) were transfected with a scrambled, nonsilencing sequence as previously described [22].

### 4.2. Western Blot and Immunoprecipitation

Western blot was performed as described with antibodies against EDF1 (AVIVA Systems Biology Corporation, San Diego, CA, USA), rabbit anti-PPARγ, and anti-actin (Sigma Aldrich, St. Louis, MO, USA) [15]. Secondary antibodies were labeled with horseradish peroxidase (GE Healthcare, Milano, Italy). The immunoreactive proteins were visualized with the SuperSignal chemiluminescence kit (ThermoFisher Scientific, Waltham, MA, USA). To coimmunoprecipitate EDF1 and PPARγ, lysates were immunoprecipitated using monoclonal antibodies against PPARγ. Nonimmune immunoglobulin G (IgGs) were used as controls (Supplementary S3b). After binding to protein G-Sepharose, the samples were processed for western blot with rabbit anti-EDF1 antibodies. Nuclear and cytosolic fractions were obtained as described [20] and processed by western blot using antibodies against EDF1, anti-glyceraldehyde-3-phosphate dehydrogenase (GAPDH), and anti-TATA Binding Protein (TBP) (Santa Cruz Biotechnology-Tebu Bio, Huissen, The Netherlands). All the experiments were repeated at least three times. One representative blot is shown in the figures. Densitometry was performed using ImageJ software (1.50i, National Institutes of Health, Bethesda, MD, USA) on three blots and expressed using an arbitrary value scale. Results are shown as the mean ± standard deviation of three separate experiments.

### 4.3. Immunofluorescence Staining

Subconfluent HUVEC on gelatin-coated coverslips were treated or not treated with VEGF (50 ng/mL) (PeproTech, London, UK), fixed in phosphate-buffered saline containing 3% paraformaldehyde and 2% sucrose pH 7.6, permeabilized with HEPES-Triton 1%, incubated with anti-EDF1 immunopurified IgGs, and stained with rhodamine-conjugated anti-rabbit IgGs [20]. Staining with rabbit nonimmune IgGs did not yield any significant signal.

### 4.4. Reporter Gene Assay

To study PPARγ activity, subconfluent HUVEC with silenced *EDF1* and their controls were transfected with plasmids pDR1-Luc (0.2 μg/cm^2^), using Arrest-in transfection reagent (Invitrogen) as described [24]. Luciferase activity was measured after 24 h of treatment with VEGF (50 ng/mL) using a luminometer. The transfection efficiency was normalized against a cotransfected reporter plasmid phRL-TK encoding Renilla luciferase (5 ng/cm^2^), by dividing the firefly luciferase activity by the Renilla luciferase activity according to the Dual-Luciferase Reporter Assay kit manual (Promega, Madison, WI, USA). The experiments were performed in triplicate and the results are shown as the mean ± standard deviation of three separate experiments.

### 4.5. Real-Time-PCR

Total RNA was extracted using the PureLink RNA Mini kit (Ambion, Thermo Fisher Scientific, Waltham, MA, USA). After quantification, equivalent amounts of total RNA were assayed by first strand cDNA synthesis using SuperScript II RT (Invitrogen, Carlsbad, CA, USA). Real-time PCR was performed at least two times in triplicate on the 7500 FAST Real-Time PCR System instrument using TaqMan Gene Expression Assays (Life Technologies, Monza, Italy). We analyzed *FABP4* (Hs01086177_m1), *EDF1* (Hs00610152_m1), and *PPARγ* (Hs01115513_m1) while the housekeeping gene *GAPDH* (Hs99999905_m1) was used as an internal reference gene. Relative changes in gene expression were analyzed by the 2^−ΔΔCt^ method.

### 4.6. NO Release

Griess assay was used to measure NO in cell culture media [23]. In particular, media were mixed 1:1 with fresh Griess solution and the absorbance was measured at 550 nm. The concentration of nitrites in the media were determined using calibration curve generated using known concentration of NaNO_2_ solutions. The experiment was performed in triplicate and the results are shown as the mean ± standard deviation of three separate experiments.

### 4.7. Statistical Analysis

Statistical significance was determined using the Student’s *t* test and set at *p* values less than 0.05. In the figures, * *p* < 0.05, ** *p* < 0.01, *** *p* < 0.001.

## Supplementary Materials

Supplementary materials can be found at http://www.mdpi.com/1422-0067/19/7/1830/s1.

## Abbreviations

VEGF	Vascular Endothelial Growth Factor
EDF	Endothelial Differentiation-related factor
PPARγ	Peroxisome proliferator-activated receptor γ
EC	Endothelial cells
NO	Nitric oxide

## References

[1] Duan S.Z., Usher M.G., Mortensen R.M. (2008). Peroxisome Proliferator-Activated Receptor—Mediated Effects in the Vasculature. Circ. Res..

[2] Chawla A., Schwarz E.J., Dimaculangan D.D., Lazar M.A. (1994). Peroxisome proliferator-activated receptor (PPAR) γ: Adipose predominant expression and induction early in adipocyte differentiation. Endocrinology.

[3] Ahmadian M., Suh J.M., Hah N., Liddle C., Atkins A.R., Downes M., Evans R.M. (2013). PPARgamma signaling and metabolism: The good, the bad and the future. Nat. Med..

[4] Imai T., Takakuwa R., Marchand S., Dentz E., Bornert J.M., Messaddeq N., Wendling O., Mark M., Desvergne B., Wahli W. (2004). Peroxisome proliferator-activated receptor γ is required in mature white and brown adipocytes for their survival in the mouse. Proc. Natl. Acad. Sci. USA.

[5] Tomaru T., Steger D.J., Lefterova M.I., Schupp M., Lazar M.A. (2009). Adipocyte specific expression of murine resistin is mediated by synergism between peroxisome proliferator-activated receptor γ and CCAAT/enhancer-binding proteins. J. Biol. Chem..

[6] Iwaki M., Matsuda M., Maeda N., Funahashi T., Matsuzawa Y., Makishima M., Shimomura I. (2003). Induction of adiponectin, a fat-derived antidiabetic and antiatherogenic factor, by nuclear receptors. Diabetes.

[7] Halabi C.M., Beyer A.M., de Lange W.J., Keen H.L., Baumbach G.L., Faraci F.M., Sigmund C.D. (2008). Interference with PPARγ Function in Smooth Muscle Causes Vascular Dysfunction and Hypertension. Cell Metab..

[8] Kotlinowski J., Jozkowicz A. (2016). PPAR Gamma and Angiogenesis: Endothelial Cells Perspective. J. Diabetes Res..

[9] Kleinhenz J.M., Kleinhenz D.J., You S., Ritzenthaler J.D., Hansen J.M., Archer D.R., Sutliff R.L., Hart C.M. (2009). Disruption of endothelial peroxisome proliferator-activated receptor-γ reduces vascular nitric oxide production. Am. J. Physiol. Heart Circ. Physiol..

[10] Chen Z., Ishibashi S., Perrey S., Osuga J., Gotoda T., Kitamine T., Tamura Y., Okazaki H., Yahagi N., Iizuka Y. (2001). Troglitazone inhibits atherosclerosis in apolipoprotein E-knockout mice: Pleiotropic effects on CD36 expression and HDL. Arterioscler. Thromb. Vasc. Biol..

[11] Charbonnel B., Dormandy J., Erdmann E., Massi-Benedetti M., Skene A., PROactive Study Group (2004). The prospective pioglitazone clinical trial in macrovascular events (PROactive): Can pioglitazone reduce cardiovascular events in diabetes? Study design and baseline characteristics of 5238 patients. Diabetes Care.

[12] Wang S., Dougherty E.J., Danner R.L. (2016). PPARγ signaling and emerging opportunities for improved therapeutics. Pharmacol. Res..

[13] Rosenfeld M.G., Lunyak V.V., Glass C.K. (2005). Sensors and signals: A coactivator/corepressor/epigenetic code for integrating signal-dependent programs of transcriptional response. Genes Dev..

[14] Brendel C., Gelman L., Auwerx J. (2002). Multiprotein bridging factor-1 (MBF-1) is a cofactor for nuclear receptors that regulate lipid metabolism. Mol. Endocrinol..

[15] Leidi M., Mariotti M., Maier J.A. (2009). Transcriptional coactivator EDF-1 is required for PPARgamma-stimulated adipogenesis. Cell. Mol. Life Sci..

[16] Takemaru K., Li F.Q., Ueda H., Hirose S. (1997). Multiprotein bridging factor 1 (MBF1) is an evolutionarily conserved transcriptional coactivator that connects a regulatory factor and TATA element-binding protein. Proc. Natl. Acad. Sci. USA.

[17] Busk P.K., Wulf-Andersen L., Strøm C.C., Enevoldsen M., Thirstrup K., Haunsø S., Sheikh S.P. (2003). Multiprotein bridging factor 1 cooperates with c-Jun and is necessary for cardiac hypertrophy in vitro. Exp. Cell Res..

[18] Liu Q.X., Jindra M., Ueda H., Hiromi Y., Hirose S. (2003). Drosophila MBF1 is a co-activator for Tracheae Defective and contributes to the formation of tracheal and nervous systems. Development.

[19] Dragoni I., Mariotti M., Consalez G.G., Soria M.R., Maier J.A. (1998). EDF-1, a novel gene product down-regulated in human endothelial cell differentiation. J. Biol. Chem..

[20] Ballabio E., Mariotti M., De Benedictis L., Maier J.A. (2004). The dual role of endothelial differentiation-related factor-1 in the cytosol and nucleus: Modulation by protein kinase A. Cell. Mol. Life Sci..

[21] Bautch V.L. (2012). VEGF-Directed Blood Vessel Patterning: From Cells to Organism. Cold Spring Harb. Perspect. Med..

[22] Mariotti M., De Benedictis L., Avon E., Maier J.A.M. (2000). Interaction between endothelial differentiation-related factor-1 and calmodulin in vitro and in vivo. J. Biol. Chem..

[23] Leidi M., Mariotti M., Maier J.A. (2010). EDF-1 contributes to the regulation of nitric oxide release in VEGF-treated human endothelial cells. Eur. J. Cell Biol..

[24] Castiglioni S., Cazzaniga A., Maier J.A. (2014). Potential interplay between NFκB and PPARγ in human dermal microvascular endothelial cells cultured in low magnesium. Magnes. Res..

[25] Furuhashi M., Saitoh S., Shimamoto K., Miura T. (2015). Fatty Acid-Binding Protein 4 (FABP4): Pathophysiological Insights and Potent Clinical Biomarker of Metabolic and Cardiovascular Diseases. Clin. Med. Insights Cardiol..

[26] Elmasri H., Ghelfi E., Yu C.W., Traphagen S., Cernadas M., Cao H., Shi G.P., Plutzky J., Sahin M., Hotamisligil G. (2012). Endothelial cell-fatty acid binding protein 4 promotes angiogenesis: Role of stem cell factor/c-kit pathway. Angiogenesis.

[27] Magri C.J., Gatt N., Xuereb R.G., Fava S. (2011). Peroxisome proliferator-activated receptor-γ and the endothelium: Implications in cardiovascular disease. Expert Rev. Cardiovasc. Ther..

[28] Aragonès G., Saavedra P., Heras M., Cabré A., Girona J., Masana L. (2012). Fatty acid-binding protein 4 impairs the insulin-dependent nitric oxide pathway in vascular endothelial cells. Cardiovasc. Diabetol..

[29] Gheibi S., Jeddi S., Kashfi K., Ghasemi A. (2018). Regulation of vascular tone homeostasis by NO and H_2_S: Implications in hypertension. Biochem. Pharmacol..

[30] Cho D.H., Choi Y.J., Jo S.A., Jo I. (2004). Nitric oxide production and regulation of endothelial nitric-oxide synthase phosphorylation by prolonged treatment with troglitazone: Evidence for involvement of peroxisome proliferator-activated receptor (PPAR) gamma-dependent and PPARgamma-independent signaling pathways. J. Biol. Chem..

