# Supplementary material for: The Contribution of EDF1 to PPARγ Transcriptional Activation in VEGF-Treated Human Endothelial Cells
Source: Int J Mol Sci. 2018 Jun 21;19(7):1830. doi: 10.3390/ijms19071830 (PMC6073190; doi:10.3390/ijms19071830)
Supplement: Supplementary file 1 [file ijms-19-01830-s001.docx]

**Supplementary: EDF-1 contributes to PPARγ transcriptional activation in VEGF- treated human endothelial cells**

**Alessandra Cazzaniga^1†^, Laura Locatelli^1†^, Sara Castiglioni^1^, and Jeanette Maier^1*^**

**Figure S1.** The total amounts of EDF-1 and PPARγ. Western blot with antibodies against EDF-1 and PPARγ. Densitometric analysis was performed by the ImageJ software and EDF-1 or PPARγ /actin ratio was calculated on three blots from separate experiments ± standard deviation.

**
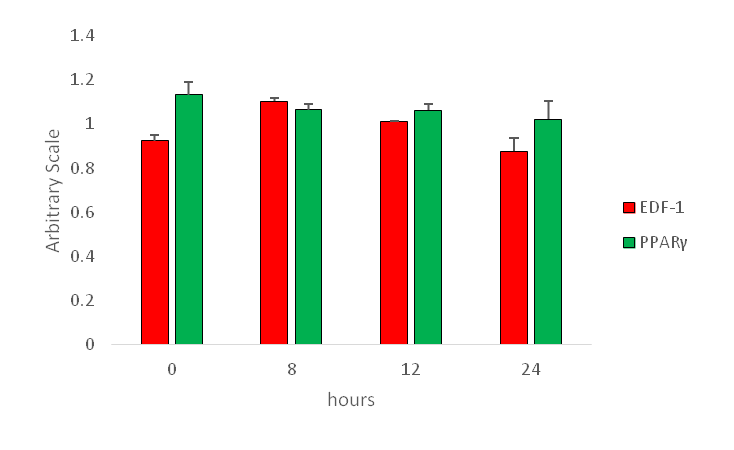
**

**Figure S2.** Subcellular localization of EDF-1 in cells treated VEGF. (a) Fluorescence intensity was measured using the ImageJ software and a representative graph was made comparing the intensity of fluorescence of CTR 1h (100%) to the other samples. (b) Western blot was performed on nuclear and cytosolic fractions using antibodies against EDF-1, GAPDH and TBP. Densitometric analysis was performed by the ImageJ software. EDF-1/GAPDH and EDF-1/TBP ratio were calculated for cytosolic and nuclear fractions respectively, on three blots from separate experiments ± standard deviation.

**
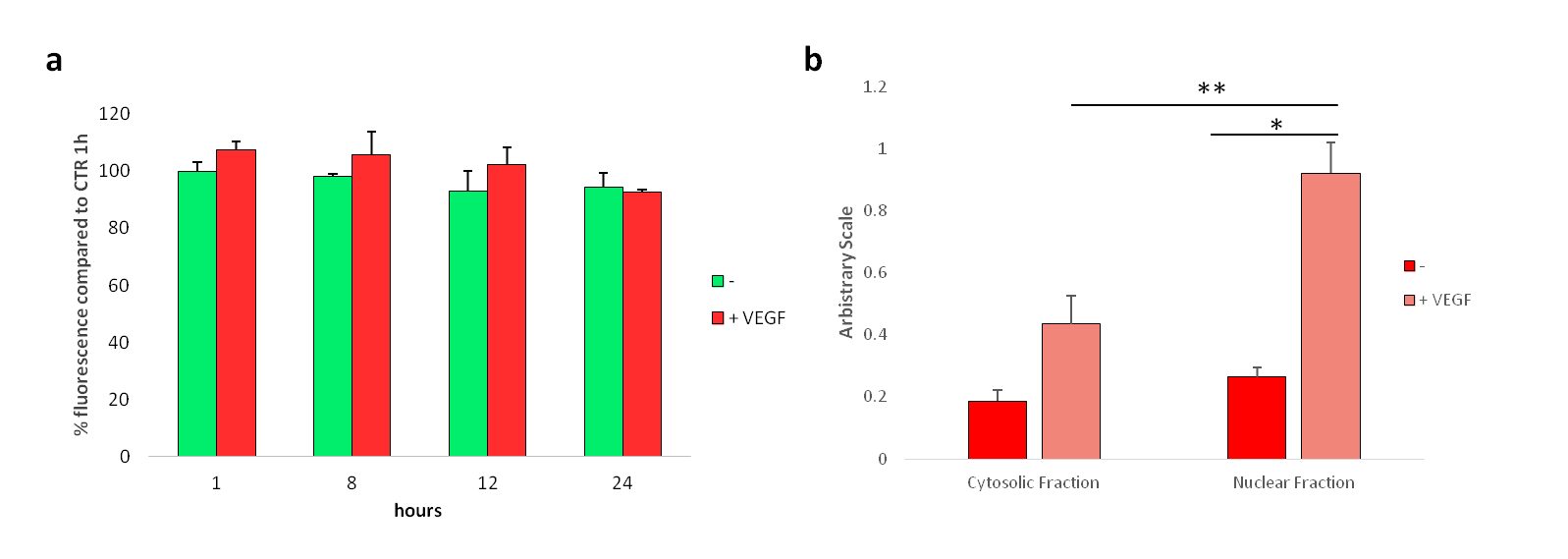
**

**Figure S3. The interaction between EDF-1 and PPARγ in HUVEC treated VEGF.** The whole blots of immunoprecipitation are reported (a). Cell lysates were immunoprecipitated with non immune IgGs, and blotted with antibodies against EDF-1 (upper panel) or PPARγ (lower panel) (b).

**
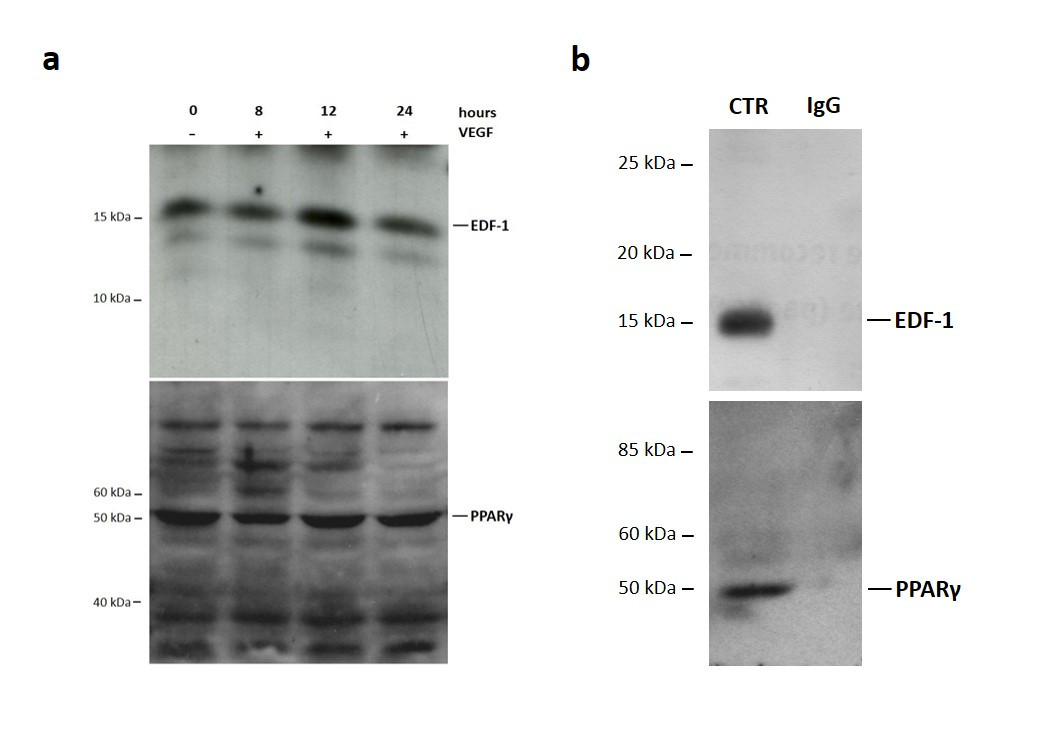
**.

**Figure S4**. PPARγ transcriptional activity in HUVEC silencing EDF-1. Western blot using antibodies against EDF-1 and PPARγ was performed on HUVEC and αs1. Densitometric analysis was performed by the ImageJ software and EDF-1 or PPARγ /actin ratio was calculated on three blots from separate experiments ± standard deviation.

**
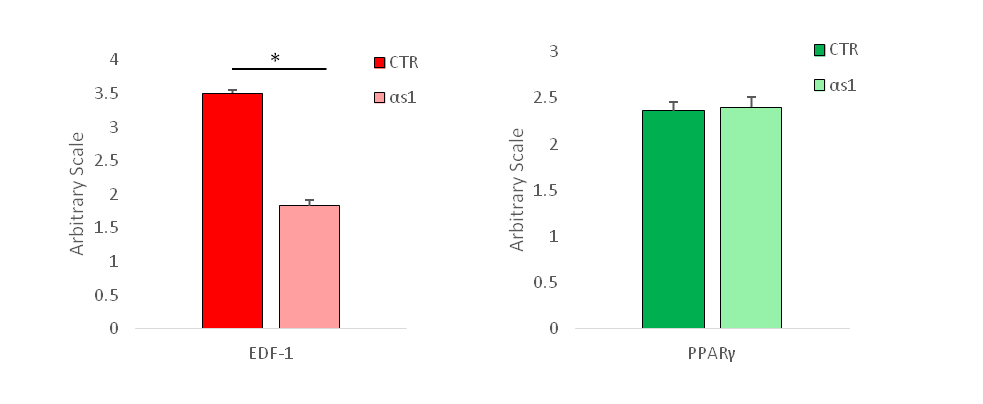
**
